# Unique structural attributes of the PSI‐NDH supercomplex in *Physcomitrium patens*

**DOI:** 10.1111/tpj.17116

**Published:** 2024-11-03

**Authors:** Monika Opatíková, Roman Kouřil

**Affiliations:** ^1^ Department of Biophysics, Faculty of Science Palacký University Olomouc Czech Republic

**Keywords:** PSI‐NDH supercomplex, transmission electron microscopy, single particle analysis, *Physcomitrium patens*, LHCA5, cyclic electron transport

## Abstract

Cyclic electron transport around photosystem I (PSI) is essential for the protection of the photosynthetic apparatus in plants under diverse light conditions. This process is primarily mediated by Proton Gradient Regulation 5 protein/Proton Gradient Regulation 5‐like photosynthetic phenotype 1 protein (PGR5/PGRL1) and NADH dehydrogenase‐like complex (NDH). In angiosperms, NDH interacts with two PSI complexes through distinct monomeric antennae, LHCA5 and LHCA6, which is crucial for its higher stability under variable light conditions. This interaction represents an advanced evolutionary stage and offers limited insight into the origin of the PSI‐NDH supercomplex in evolutionarily older organisms. In contrast, the moss *Physcomitrium patens* (*Pp*), which retains the *lhca5* gene but lacks the *lhca6*, offers a glimpse into an earlier evolutionary stage of the PSI‐NDH supercomplex. Here we present structural evidence of the *Pp* PSI‐NDH supercomplex formation by single particle electron microscopy, demonstrating the unique ability of *Pp* to bind a single PSI in two different configurations. One configuration closely resembles the angiosperm model, whereas the other exhibits a novel PSI orientation, rotated clockwise. This structural flexibility in *Pp* is presumably enabled by the variable incorporation of LHCA5 within PSI and is indicative of an early evolutionary adaptation that allowed for greater diversity at the PSI‐NDH interface. Our findings suggest that this variability was reduced as the structural complexity of the NDH complex increased in vascular plants, primarily angiosperms. This study not only clarifies the evolutionary development of PSI‐NDH supercomplexes but also highlights the dynamic nature of the adaptive mechanisms of plant photosynthesis.

## Significance Statement

The moss *Physcomitrium patens* expresses unique structural variability in the formation of PSI‐NDH supercomplex, responsible for cyclic electron transport around photosystem I. Analysis of *P. patens* PSI‐NDH structure provides new insights into the gradual evolution of this supercomplex in terrestrial plants.

## INTRODUCTION

In the initial phase of oxygenic photosynthesis, the energy of absorbed light powers the electron transport chain within thylakoid membranes (TM) of plants, algae and cyanobacteria. This process involves water and NADP^+^ as the primary electron donor and final electron acceptor, respectively, as reviewed, for example, in Allen (2002). In addition to the characteristic linear electron transport, photosynthetic organisms are able to initiate cyclic electron transport (CET) around photosystem I (PSI) when it is necessary to increase the ATP/NADPH production ratio and/or to protect the photosynthetic machinery from environmental stresses such as extreme, low or variable light conditions. The major mediator of CET is the antimycin‐A‐sensitive Proton Gradient Regulation 5 protein/Proton Gradient Regulation 5‐like photosynthetic phenotype 1 protein (PGR5/PGRL1) (DalCorso et al., 2008; Hertle et al., 2013; Munekage et al., 2002; Sugimoto et al., 2013; Tagawa et al., 1963). The pathway mediated by the NADH dehydrogenase‐like complex (hereafter NDH), classified as a minor contributor to CET, usually shows its importance under stress conditions such as (i) low light (Ueda et al., 2012; Yamori et al., 2011, 2015), (ii) fluctuating light (Basso et al., 2020; Niu et al., 2024; Ueda et al., 2012; Yamori et al., 2016) or (iii) far‐red light (Kono & Terashima, 2016). The nomenclature ‘like’ for this complex is based on its partial gene homology with respiratory complex I (see, e.g. Ifuku et al., 2011). However, unlike its respiratory counterpart, the chloroplast NDH transfers electrons from bound ferredoxin (Fd) instead of NADH (see, e.g. Schuller et al., 2019).

The NDH is a multi‐subunit complex whose composition is closely related to the evolutionary position of the species, and while in cyanobacteria, it can contain at least 19 subunits (Laughlin et al., 2019), in angiosperms, it has a more complex arrangement with at least 29 subunits (Shikanai, 2016). Based on function, the NDH complex can be divided into the following five subcomplexes: (i) the SubM subcomplex (the membrane‐embedded subunits NdhA–NdhG), the stromal hydrophilic arm formed by (ii) SubA (NdhH–NdhO), (iii) the electron donor‐binding subcomplex SubE (NdhS–NdhV), (iv) SubB (PnsB1–PnsB5) and (v) a lumenal subcomplex, SubL (PnsL1–PnsL5). The last two subcomplexes are unique to the chloroplast NDH complex (Armbruster et al., 2013; Ifuku et al., 2011), when the SubL is found exclusively in angiosperms (Shikanai, 2016). However, these two subcomplexes are not fully developed in evolutionary older land plants, in which several subunits of SubB and SubL are represented only by their functional orthologues (Ueda et al., 2012). Moreover, these orthologues still lack key genetic modifications and therefore cannot participate in the NDH formation (Ifuku et al., 2011; Shikanai, 2016, 2020).

In vascular plants, the structural and related functional stability of the NDH complex is critically dependent on its interaction with the PSI complex. PSI is primarily composed of a core and a light‐harvesting complex (LHCI), which includes four LHCI proteins (LHCA1–4) that form a characteristic half‐belt around the PSI core (e.g. Amunts et al., 2010; Mazor et al., 2015). It was found that the interaction between NDH and the PSI complex occurs only in the presence of two additional light‐harvesting proteins, LHCA5 and/or LHCA6 (Peng et al., 2009). The evolution of the LHCA5 protein in land plants, and additionally, LHCA6 in angiosperms is thought to have been caused by the need of plants to stabilise the NDH complex in a terrestrial light environment (Kato, Odahara, et al., 2018; Peng & Shikanai, 2011). While a closer examination of the evolutionary distribution of LHCA5 reveals that a functional orthologue of LHCA5 is already present in moss (Iwai & Yokono, 2017; Kato, Odahara, et al., 2018), its distribution is not consistent across species. For example, genes encoding LHCA5 are absent in some gymnosperms, such as Pinales and Gnetales, or in the liverwort *Marchantia polymorpha* (Grebe et al., 2019; Ueda et al., 2012).

Single particle electron microscopy (EM) analysis showed that the interaction between the NDH complex and PSI is mediated by LHCI. In barley, two copies of PSI bind along the membrane part of the curved NDH complex (Kouřil et al., 2014). Recent cryo‐EM studies on two angiosperm species, barley and *Arabidopsis thaliana* (*At*), provided a detailed characterisation of the PSI‐NDH supercomplex (sc) (Shen et al., 2022; Su et al., 2022). The solved structures, both expressing a high level of structural similarity, confirmed the crucial roles of the LHCA5 and LHCA6 proteins, each mediating the binding of one of the two PSI complexes at the opposite sites of the membrane part of NDH complex. The stronger interaction with the NDH complex is mediated by a stromal loop of LHCA6 from one PSI, functionally replacing LHCA2, which interacts with the NdhF subunit of SubM and the PnsB2 and PnsB5 subunits of SubB at the outer part of the curved membrane domain. Conversely, the weaker, yet evolutionary pioneering interaction, involves LHCA5 from the second PSI, functionally substituting for LHCA4, and interacting with the PnsB1 subunit of the SubB subcomplex at the inner part of the curved membrane domain. The attributed weaker interaction via LHCA5 is based on observations of (i) the easier disconnection of the NDH‐bound PSI complex by LHCA5 (e.g. Kouřil et al., 2014), (ii) a larger gap and fewer interacting partners between NDH and LHCA5 in comparison with NDH‐LHCA6 (Su et al., 2022) and (iii) the presence of a fraction of free PSI with bound LHCA5 but lacking LHCA6, as the latter remains strongly bound to NDH (Peng et al., 2009). These findings highlight the evolutionary significance of the SubB and SubL subdomains, which facilitated the formation of the PSI‐NDH sc, since the formation of this sc plays a key role in the stability and function of NDH, particularly under higher light intensities (Armbruster et al., 2013; Ifuku et al., 2011; Pan et al., 2020; Schuller et al., 2019; Shikanai, 2016, 2020).

*Physcomitrium* (formerly *Physcomitrella*) *patens* (*Pp*) is a model organism for non‐seed, non‐vascular plants belonging to the Bryophyta group, which includes mosses, liverworts and hornworts (Rensing et al., 2008). Focusing on its PSI, *Pp* is notable for its redundancy in the *lhca1–3* gene paralogues in contrast to the single‐encoded *lhca1–3* found in green algae and vascular plants. Moreover, although *Pp* encodes a functional orthologue of the *At lhca5* gene, it lacks the *lhca4* and *lhca6* genes (Alboresi et al., 2008; Busch et al., 2013; Iwai & Yokono, 2017; Rensing et al., 2008; Zimmer et al., 2013). Despite these genetic differences, *Pp* is still able to form ‘typical form’ of PSI complex with its characteristic LHCI belt, although with slight variation in antennae composition, as revealed by Gorski et al. (2022). In their high‐resolution structure of *Pp* PSI, the substituting antenna for LHCA4 was identified as the unique *Pp* LHCA2b paralogue. With the rest of the antennae comparable to those in vascular plants the arrangement of the LHCI belt in *Pp* is as follows: LHCA1‐LHCA2b‐LHCA2a‐LHCA3. This finding contradicts the results from Yan et al. (2021), who identified LHCA5 as the substituent for LHCA4. In contrast to vascular plants and comparably to *Chlamydomonas reinhardtii* (*Cr*), *Pp* is able to form larger PSI complexes under low light conditions, where the LHCI antenna is composed of two rows of LHCA proteins, including LHCII trimer and the LHCB9 protein (Iwai et al., 2015, 2018; Pinnola et al., 2018), the last one unique only to *Pp* (Alboresi et al., 2008). However, while the outer belt of LHCA proteins in *Cr* follows the position of its inner belt (Qin et al., 2019; Suga et al., 2019), the outer belt in *Pp* is significantly shifted counterclockwise towards LHCB9 (Sun et al., 2023; Zhang et al., 2023).

The absence of *lhca4* and *lhca6* genes, as well as genes encoding the PnsB2 and PnsB3 subunits of SubB in *Pp* (Alboresi et al., 2008; Busch et al., 2013; Iwai & Yokono, 2017; Kato et al., 2021), raises important questions about the formation and architecture of the PSI‐NDH sc in this organism (Iwai & Yokono, 2017; Kato, Odahara, et al., 2018; Storti et al., 2020). Although biochemical analysis supports the existence of PSI‐NDH formation in *Pp* (Kato, Odahara, et al., 2018), direct visual evidence is still lacking. In our study, we focused on the isolation and structural characterisation of this putative PSI‐NDH sc in *Pp*. Single particle EM analysis confirmed that *Pp* is indeed capable of forming this sc. Moreover, our results uncovered an unusual variability in the binding of PSI to NDH, which is likely related to LHCA5 binding at two different positions within the LHCI belt. This variability, not yet observed in vascular plants, offers new insights into the diversity of interactions between PSI and NDH, leading to variable formations of the PSI‐NDH sc across evolutionarily distinct plant species.

## RESULTS

### Separation and structural characterisation of the PSI‐NDH supercomplex

**Figure 1:**
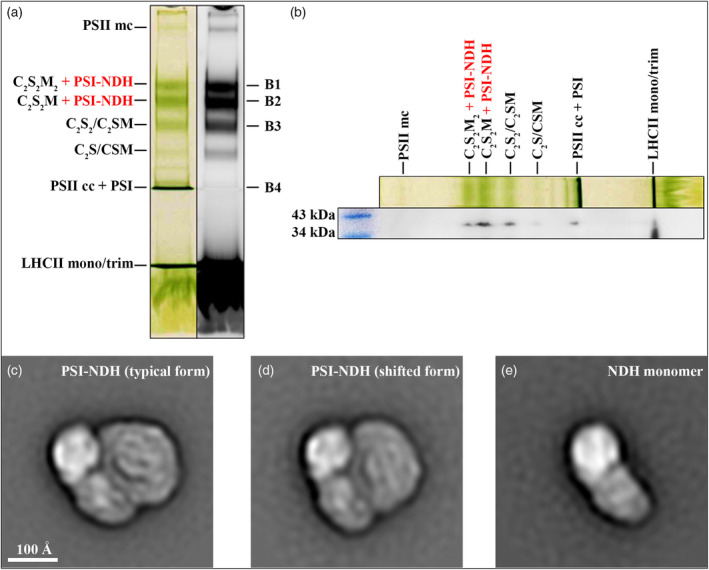
Separation and identification of pigment–protein complexes from *Physcomitrium patens* and structural characterisation of PSI‐NDH/NDH by single particle electron microscopy. (a) CN‐PAGE separation of pigment–protein complexes from the TM from *Pp* solubilised by 0.5% β‐DDM. The image shows the selected gel line scanned at room temperature in transmission mode (colour image) and in fluorescence mode (black and white image). (b) Immunoblot identification of the NdhH subunit on a PVDF membrane with transferred proteins from the second‐dimension denaturing PAGE. Two horizontal bands of the marker (blue colour) designate the molecular weight of proteins at these positions. (c–e) Electron density maps of two forms of PSI‐NDH sc and NDH monomer from *Pp* revealed by single particle electron microscopy (stromal view). The 2D class average of (c) the typical form of PSI‐NDH sc consisting of 2566 particles, (d) the shifted form consisting of 1098 particles, and (e) the NDH monomer composed of 2725 particles. B1, C_2_S_2_M_2_ band; B2, C_2_S_2_M band; B3, C_2_S_2_/C_2_SM band; B4, PSII cc + PSI band; β‐DDM, β‐D‐maltoside; cc, core complex, CN‐PAGE, Clear‐Native Polyacrylamide Gel Electrophoresis; LHCII, light‐harvesting complex of photosystem II; mc, megacomplex; mono, monomer; *Pp*, *Physcomitrium patens*; PSI, photosystem I; PSII, photosystem II; TM, thylakoid membrane; trim, trimer.

Preparation of the PSI‐NDH sample for transmission EM was preceded by solubilisation of the isolated TM with the non‐ionic detergent *n*‐dodecyl β‐D‐maltoside (β‐DDM) and separation of protein complexes by Clear‐Native Polyacrylamide Gel Electrophoresis (CN‐PAGE). The separated proteins in gel expressed a characteristic pattern highly similar to the pattern observed for vascular plants (Figure 1a). To determine the relative distribution and abundance of NDH‐containing sc/NDH monomer within specific bands, we performed second‐dimension electrophoresis of the 1D gel line under denaturing conditions, followed by a Western blot using an antibody against the NdhH subunit (Figure 1b). Because the predicted molecular mass of the sc containing one copy of each, NDH and the PSI complex, in *Pp* is approx. 1300 kDa, we expected its highest abundance to be in close proximity to the two largest photosystems II (PSII) sc bands represented by the C_2_S_2_M_2_ and C_2_S_2_M forms, having approx. 1400 and 1250 kDa, respectively. This premise was confirmed by immunoblot analysis, which revealed a specific signal for the NdhH subunit at the position of both bands, with the strongest signal observed at the band with lower molecular weight. However, we also identified weaker signals for the NdhH subunit at the positions of the C_2_S_2_/C_2_SM and C_2_S/CSM bands, at the band with PSII core complex/PSI complex and at the level of LHCII monomer/trimer band. The two signals observed in the abovementioned smaller PSII sc forms likely result from the gradual disintegration of the PSI‐NDH sc and the disconnection of its most flexible subunits, which may also explain the partially split antibody signal at the C_2_S_2_M position. The signal localised at the position of the PSII core complex/PSI complex band would correspond to the expected position of NDH monomer in *Pp* with a molecular weight of approx. 670–700 kDa. The signal at the position of LHCII monomers and trimers most likely represents the individual NdhH subunits. Notably, no NdhH signal was detected above the C_2_S_2_M_2_ band, which is in agreement with the inability of *Pp* to bind more than one PSI to NDH. Based on the immunoblot results, we selected three bands with the highest abundance of potential PSI‐NDH sc (C_2_S_2_M_2_, C_2_S_2_M and C_2_S_2_/C_2_SM hereafter referred to as B1, B2, and B3, respectively), which were individually processed for subsequent EM analysis after spontaneous elution from the excised gel pieces.

EM inspection of specimens prepared from individual bands B1, B2 and B3 revealed the presence of PSI‐NDH scs only in the B1 and B2 bands, while the corresponding forms of PSII scs were highly abundant in all three bands. It is also evident that the relative abundance of PSI‐NDH scs to PSII scs was several times higher in the B2 band than in the B1 band (Figure S1a–c). The micrographs of specimens from the B1 and B2 bands also showed a minor presence of individual NDH complexes, which is probably due to the disintegration of the PSI‐NDH sc during gel elution, as a result of weak interaction mediated by LHCA5. EM analysis of the B3 band did not reveal the presence of PSI‐NDH scs or NDH complexes, despite the positive immunodetection of the NdhH subunit in this band, probably due to their very low concentration. Image analysis of single particle projections of the PSI‐NDH scs and NDH complexes revealed their 2D structural arrangement in *Pp* (Figure 1c–e; Figure S1d). PSI‐NDH sc consists of a single PSI complex that binds to the inner part of the curved membrane arm of the NDH. Surprisingly, the classification of PSI‐NDH sc led to the discovery of two distinct types of this sc (Figure 1c,d). These two forms differ in the orientation of bound PSI relative to NDH. The more abundant form of PSI‐NDH sc in our data set (Figure 1c) closely resembled its counterpart observed in barley and *At*. However, in the second, less abundant form, the position of PSI was shifted (Figure 1d), suggesting a change in the interaction between NDH and PSI.

**Figure 2:**
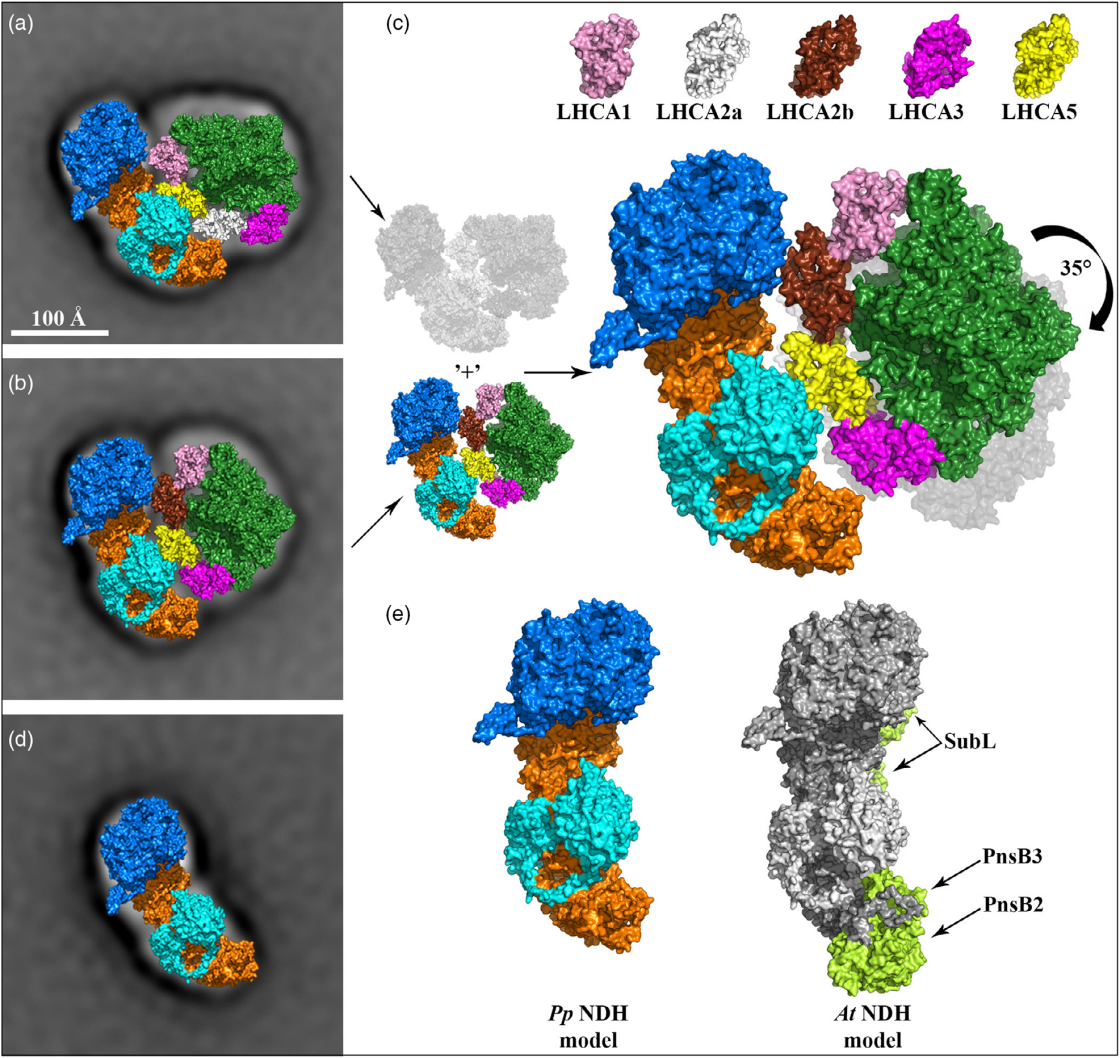
Structural characterisation of PSI‐NDH supercomplex and NDH monomer in *Physcomitrium patens*. (a) Structural model of the PSI‐NDH sc in *Pp*, which corresponds to the arrangements in the PSI‐NDH sc from *At*. (b) Structural model of the PSI‐NDH sc, where PSI is rotated clockwise by 35° in comparison to its position in a. (c) Superposition of the *Pp* PSI‐NDH model with the rotated PSI from image b (in colour) over its unrotated form from a (grey colour) with pictorial colour‐coded legend of individual LHCA antennae. (d) Structural model of the NDH monomer fitted by the truncated *At* NDH. (e) Comparison of the *Pp* NDH model (in colour) versus the complete *At* NDH model (grey) with highlighted subunits absent in *Pp* NDH (limon). Structural models of PSI‐NDH scs viewed from the stromal side were obtained by a fit of the NDH monomer from *At* (PDB ID: 7WG5, Su et al., 2022) and PSI complex from *Pp* (PDB ID: 7KSQ, Gorski et al., 2022). Prior to fitting, the subunits of NDH unencoded in *Pp* genome were removed from the *At* NDH structure, namely the PnsB2, PnsB3 and the subunits of SubL. PSI subunits and NDH subcomplexes and selected subunits are colour‐coded. PSI: forest green—core; pink, white, chocolate, yellow and magenta—LHCA1, LHCA2a, LHCA2b, LHCA5 and LHCA3, respectively. NDH: orange—SubM, cyan—SubB (PnsB1, PnsB4, PnsB5), blue—SubA and NdhT, lime—SubB (PnsB2, PnsB3) and SubL.

To interpret the EM projection maps of the PSI‐NDH scs, they were fitted with structural model of PSI complex from *Pp* (PDB ID: 7KSQ, Gorski et al., 2022) and NDH from *At*, where the subunits not encoded in the *Pp* genome, namely PnsB2, PnsB3 and subcomplex SubL, were removed from the model (PDB ID: 7WG5, Su et al., 2022). The constructed structural model of the more abundant *Pp* PSI‐NDH sc suggests that the position and orientation of PSI relative to NDH is equivalent to that observed in *At* and barley (Figure 2a). This arrangement indicates a typical interaction that is mediated by the LHCA5 antenna of PSI, which replaces the LHCA4 antenna, and PnsB1 subunit of NDH (Shen et al., 2022; Su et al., 2022). However, in the case of *Pp*, which is characterised by the absence of LHCA4, LHCA5 replaces the LHCA2b paralogue in an identical position. In contrast, the structural model of the less represented *Pp* PSI‐NDH sc reveals a different interaction between PSI and NDH, due to the rotation of PSI with respect to NDH (Figure 2b). By superposing the two obtained models of *Pp* PSI‐NDH scs, we found that the PSI in the latter model is rotated clockwise by approx. 35° compared to the position of PSI in the typical structure, corresponding to its rotation by one LHCA protein (Figure 2c). At first glance, this rotation pattern would therefore indicate a PSI interaction mediated through the neighbouring LHCA2a antenna, assuming a conserved arrangement of antennae in the LHCI belt of PSI. However, since LHCA5 was shown to be essential for the formation of PSI‐NDH sc in *Pp* (Kato, Odahara, et al., 2018), we assume that LHCA2a was substituted by LHCA5 in this case as well (Figure 2b). This apparently allows *Pp* to form two distinct types of PSI‐NDH scs, depending on whether LHCA5 replaces either LHCA2a or LHCA2b.

Consistent with the densities of *Pp* PSI‐NDH scs, the EM projection map of the free *Pp* NDH monomer was successfully fitted with the modified *At* NDH model (Figure 2d). Comparison of this truncated *Pp* NDH model with the complete *At* NDH model shows increased model complexity in angiosperms due to the addition of the PnsB2 and PnsB3 subunits and the luminal SubL subcomplex (Figure 2e).

### Proteomic analysis of the PSI‐NDH supercomplex composition

To characterise the subunit composition of the *Pp* PSI‐NDH sc in detail, we performed a proteomic analysis by mass spectrometry (MS) on the B1, B2 and B3 bands. In order to obtain information on the composition of LHCI in the PSI complex itself, MS analysis was also performed on the B4 band (representing PSII cc + PSI). The representative subunit composition of the intact PSI‐NDH supercomplex is described based on MS analysis of the B2 band (Table S1), which was predicted to have the highest concentration of the supercomplex based on immunodetection and EM analysis. Focusing on the *Pp* NDH complex, we detected all subunits of SubM and SubA, subunits of SubB encoded in *Pp* (PnsB1, PnsB4–PnsB5) and three subunits of SubE (NdhS–NdhU). In the case of the NdhV subunit, there is no record in the protein database and therefore this subunit has not been identified.

Regarding the antennae of PSI, we confirmed the presence of LHCA5 together with nine other LHCI antennae isoforms (Table S1). Our detailed identification of the LHCA proteins showed that the LHCA1 and LHCA2a, are represented by three isoforms, LHCA3 by two isoforms, whereas LHCA2b and LHCA5 are both represented by a single isoform. It is worth noting that the LHCA antenna composition was very similar in all four analysed bands, with the only difference observed in band B4, which lacked one of the LHCA2a isoforms that also had minimal abundance in the other three bands (Table S2). The presence of LHCA5 in band B4 is not very surprising and is consistent with the identification of LHCA5 in the cryo‐EM structure of *Pp* PSI‐LHCI (Yan et al., 2021). However, considering the weak interaction between LHCA5 and NDH, in combination with the used detergents for solubilisation and the subsequent electrophoresis, we cannot exclude the possibility that the increased presence of PSI‐LHCA5 in the abovementioned band may result from PSI‐NDH sc disintegration.

In addition, MS analysis revealed the presence of the LHCB9 protein in bands B1–B3, which is crucial for the formation of larger forms of PSI (PSI‐L) in *Pp* (Iwai et al., 2018; Pinnola et al., 2018) (Table S2). The highest amount of LHCB9 was detected in band B3, consistent with a previous report showing that PSI‐L was enriched in the region of C_2_S_2_/C_2_SM PSII scs (Pinnola et al., 2018). In line with this, the amount of LHCB9 was lower in the band B1 and almost undetectable in the band B2 (Figure S2a). The fact that we did not detect the PSI‐L scs in our EM analysis is likely due to the very low abundance of all PSI‐containing scs, including PSI‐NDH, compared to other protein complexes, particularly PSII scs, as confirmed by MS analysis of bands B1, B2, and B3 (Figure S1; Figure S2b).

MS analysis provided also crucial insights into stoichiometry of LHCA5, the key protein required for the formation of the PSI‐NDH sc. The data showed that, although, LHCA5 was detected in all three bands, its abundance was negligible in band B3 (Figure S2a). This finding is in agreement with our EM analysis, which revealed the presence of projections of PSI‐NDH sc exclusively in bands B1 and B2, but none in band B3 (Figure S1). Notably, the increase in LHCA5 was primarily accompanied by a decrease in LHCA2b in bands B1 and B2, suggesting the formation of the typical form of the PSI‐NDH sc in *Pp* characteristic with LHCA5‐LHCA2b substitution (Figure 2a; Figure S2a). However, the fact that not all LHCA2b proteins were replaced by LHCA5 indicates that LHCA5 also replaces another protein, likely LHCA2a. This substitution likely leads to the formation of the shifted form of the PSI‐NDH sc (Figure 2b). However, the ambiguous distinction between the replacement of LHCA2a/2b by LHCA5 in the MS data is likely due to the simultaneous presence of the abovementioned PSI‐L supercomplex, particularly in band B1, which biases the representation of individual LHCA forms in the analysed bands. Finally, MS data allowed us to estimate the relative content of NDH and LHCA5 protein in bands B1 and B2. The slight decrease in the NDH/LHCA5 ratio in band B2 compared to band B1 suggests a higher amount of LHCA5 per NDH in band B2, indicating that more than one copy of LHCA5 is likely bound to PSI (Figure S2c), possibly replacing both LHCA2b and LHCA2a simultaneously. However, we cannot exclude the possibility of dual LHCA5 incorporation per PSI in band B1, although at lower abundance, which would result in a higher NDH/LHCA5 ratio.

## DISCUSSION

### Evolutionary adaptations of photosynthetic organisms: The role of NDH complexes and PSI‐NDH supercomplexes

As the ancestors of Bryophyta were among the first photosynthetic organisms to colonise terrestrial environments (see, e.g. Porada et al., 2016; Shaw et al., 2011), there was a significant evolutionary pressure to adapt their photosynthetic apparatus to varying light conditions. In contrast to the relatively stable, low light conditions in aquatic niches, there was a drastic increase in light intensity on land. Mosses, representing a mid‐stage of plant evolution, were one of the pioneers in land adaptation (Alboresi et al., 2008; Ifuku et al., 2011). As a result, their photosynthetic apparatus is a combination of rudimental features characteristic for algae as well as pre‐modifications that did not fully develop until angiosperms.

During the evolution of photosynthetic organisms, some mechanisms have either been repeatedly lost or have evolved independently many times in the different groups. The NDH‐mediated pathway is the mechanism that nicely fits into this frame. The *ndh* genes were lost more than once during evolution, the most prominent examples are gymnosperm family Pinaceae and several species of Gnetales (Braukmann et al., 2009; Nystedt et al., 2013), in which the loss of *ndh* genes also correlates with the loss of *lhca5* and *lhca6* genes. However, in organisms that preserved the *ndh* genes apply that, the higher in the evolution they are, the higher intricacy of this protein sc structure can be observed. A typical example would be angiosperms, in which the process of NDH assembly and its stabilisation become more complex and challenging due to the enlargement of its ‘full size version’ and therefore the evolution of additional assembling and stabilising factors become crucial (Armbruster et al., 2013; Kato, Sugimoto, & Shikanai, 2018; Peng et al., 2012; Ueda et al., 2012; Yamamoto et al., 2016, 2021). While there are indications that cyanobacteria also have the ability to form, at some level, an interaction between the PSI and NDH complex via bound phycobilisomes (Gao et al., 2016; Zhang et al., 2021), no PSI‐NDH interaction has been observed in evolutionary younger aquatic photosynthetic organisms of the green lineage and representatives of primary land plants, such as liverworts, where NDH functions as a monomer independently of its interaction with PSI. Only in vascular plants, it has been clearly demonstrated that the interaction with PSI complexes is required to further stabilise the NDH complex and prevent its disintegration, especially under high light conditions (see, e.g. Shikanai, 2016, 2020).

Initial formation of the PSI‐NDH sc was mediated by an interaction between LHCA5 of one PSI and the PnsB1 subunit of NDH. This interaction most likely originates somewhere at the level of mosses, represented by the model plant *Physcomitrium patens*, as these organisms encode both interacting proteins, LHCA5 and PnsB1 (Kato et al., 2021). In contrast, evolutionary older liverworts, as *M. polymorpha*, have lost the *lhca5* gene and are thus unable to form the characteristic PSI‐NDH sc (Ueda et al., 2012). However, the smaller version of SubB found in mosses remains sensitive to high light (Kato, Odahara, et al., 2018), suggesting that further evolutionary development of the NDH complex was required. Specifically, the expansion of the SubB subcomplex by the addition of the PnsB2 and PnsB3 subunits and the incorporation of the entire SubL subcomplex were major adaptations. Combined with the evolution of LHCA6 enabling the interaction with a second PSI, these changes increased the stability of the complex and allowed plants to adapt to higher light exposure and shift from shade to sunlit habitats (Kato et al., 2021; Otani et al., 2018; Peng et al., 2009; Shen et al., 2022; Su et al., 2022). Although the full significance and importance of these evolutionary steps are not yet fully understood, it is clear that they represent a highly coordinated and sophisticated process. This evolution increased the robustness of the PSI‐NDH sc under harsh terrestrial conditions and allowed the use of NDH‐mediated CET to regulate photosynthesis in land plants.

### Structural variability and evolutionary insights into PSI‐NDH supercomplexes in *Physcomitrium patens*

The NDH complex composition in *Pp* exhibits significant differences compared to angiosperms, primarily due to the absence of genes encoding the SubL subcomplex and the PnsB2 and PnsB3 subunits of the SubB subcomplex. Additionally, the PSI in *Pp* deviates from that in angiosperms, notably lacking the LHCA4 and LHCA6 proteins (refer to Table S1). Consequently, the absence of the LHCA6 alongside the PnsB2 and PnsB3 subunits suggests that NDH may interact with only one PSI, making *Pp* an ideal model for studying this specific configuration of the PSI‐NDH sc (Alboresi et al., 2008; Busch et al., 2013; Iwai & Yokono, 2017; Kato et al., 2021).

Our structural analysis provides direct evidence of the formation of the PSI‐NDH sc in *Pp*, revealing substantial variability in the binding of PSI to NDH. This variability is manifested by two distinct configurations of the PSI‐NDH sc (Figure 1). The predominant configuration reflects the same interaction observed between PSI and NDH in both *At* and barley (Figure 2a). In contrast, the less predominant form exhibits a unique architectural configuration that results from the different interaction between PSI and NDH (Figure 2b). This suggests a degree of structural flexibility in the assembly of the PSI‐NDH sc in *Pp*. Previously observed flexibility in PSI‐NDH structures includes the more curved NDH in *At* compared to barley (Su et al., 2022), and the slight retraction of the LHCI belt from PSI in barley (Shen et al., 2022). However, the structural variability of the PSI‐NDH sc in *Pp* is much more pronounced. As the formation of the PSI‐NDH sc in *Pp* critically depends on LHCA5, as demonstrated biochemically in a *Pp* mutant lacking this protein (Kato, Odahara, et al., 2018), our structural analysis revealed that LHCA5 presumably occupies two distinct positions in the LHCI antenna belt, at either the LHCA2b or LHCA2a site. This possibility of dual positioning leads to the formation of two distinct forms of the PSI‐NDH sc in *Pp*. Moreover, in combination with proteomic analysis, there is evidence suggesting the possibility of even double substitution, where both inner antennae LHCA2a/2b are replaced by LHCA5 simultaneously. However, this arrangement would pose a significant challenge to the regulatory mechanism responsible for selecting one specific position of LHCA5 to apply in PSI‐NDH sc formation. We hypothesise that this process would be most likely influenced by structural constraints, packing in the thylakoid membrane, or other unknown factors.

The key question, however, remains: why the ability to substitute LHCA5 for two different LHCA proteins, observed in *Pp*, has not been observed in vascular plants? We can speculate that the evolutionary elimination of different PSI‐NDH configurations in vascular plants was due to structural constraints resulting from the increasing complexity of NDH and PSI due to the incorporation of additional subunits and thus a more sophisticated PSI‐NDH sequence assembly mechanism (Kato et al., 2021; Otani et al., 2018). For instance, the PsaN subunit, which stabilises the LHCA2/LHCA3 heterodimer in the PSI of vascular plants (Amunts et al., 2010), may specifically influence the assembly of PSI‐NDH. Although this subunit does not prevent the exchange of LHCA6 for LHCA2 in the LHCI belt, thereby allowing the binding of a second PSI to NDH, its exchange mechanism may be unique to LHCA6 and inapplicable to LHCA5. Our results suggest that the absence of PsaN in *Pp* may account for the binding of LHCA5 at two positions within the LHCI belt and lead to two different PSI‐NDH sc configurations.

From a functional point of view, however, the reduction of the shifted PSI‐NDH sc form in vascular plants is not straightforward. In *Pp*, the rotation of PSI reduces the distance between the Fd‐binding sites of PSI and NDH (Figure S3), which would probably have the advantage of shortening the transport distance and increasing the local concentration of reduced Fd near NDH. Hypothetically, this closer proximity could result in higher probability of Fd binding to NDH, potentially increasing the rate of Fd re‐oxidation and overall CET effectivity. However, while the impact of this spatial arrangement on Fd reduction/oxidation remains to be confirmed, it can be assumed that the more distant PSI‐NDH arrangement conserved in vascular plants may have been compensated by the interaction of NDH with the second PSI, providing a functional advantage over the shifted PSI arrangement.

Finally, it is important to emphasise the competitive nature of PSI‐NDH formation in *Pp*, especially in the context of the formation of larger PSI complexes. These larger complexes, which include the second outer belt of LHCAs, the LHCII trimer and LHCB9 protein, forms primarily under low light conditions (Iwai et al., 2015; Pinnola et al., 2018; Sun et al., 2023; Ueda et al., 2012) and aims to increase light‐harvesting capacity (Iwai et al., 2015; Sun et al., 2023). In contrast, the PSI‐NDH sc functions in the opposite way and protects PSI from excessive light exposure (Shikanai, 2016, 2020). These opposing strategies underscore the adaptability and resilience of *Pp*, as it combines evolutionarily primitive mechanisms such as the formation of a large PSI antenna system, which is no longer necessary for most land plants, with novel functions such as stabilisation of NDH by PSI. This combination is indicative of the advanced evolutionary adaptations of *Pp*.

## MATERIALS AND METHODS

### Plant material and isolation of thylakoid membranes

Cultures of the *P. patens* subsp. *patens* (Hedwig) ecotype ‘Gransden, 2004’ were grown in the Knop medium (Reski & Abel, 1985) with added ammonium tartrate in a final concentration of 5 mM with continuous air‐bubbling in the AlgaeTron AG230 (Drásov, Czech Republic) at 80 μmol photons m^−2^ sec^−1^, 23°C, with a photoperiod 12/12 h light/dark. The 5‐days old (from the last renewal of the continuously grown culture in a ratio 1:3 for culture and new medium, respectively) plant material was harvested by simple filtration in a sieve, and the TM was immediately isolated according to the protocol described by Dau et al. (1995) with several modifications. The homogenising buffer was supplemented with the benzamidine (3 mg/100 mL) and a cocktail of protease inhibitors (cOmplete™, EDTA‐free Protease Inhibitor Cocktail, Roche, Mannheim, Germany), 2 tablets per 150 mL of buffer. All buffers used for the isolation were complemented with the phosphatase inhibitor NaF at a final concentration of 10 mM. The isolation was performed under green light and samples were kept on ice during the whole procedure. The chlorophyll content in the final TM suspension was determined spectrophotometrically by a pigment extraction in 80% acetone (Lichtenthaler, 1987).

### Clear‐native (CN‐)PAGE

The CN‐PAGE was performed according to Nosek et al. (2017) with minor modifications. TM (10 μg of chlorophyll) were solubilised with β‐DDM using a detergent: chlorophyll mass ratio of 15, respectively, and supplemented with the sample buffer [50 mM HEPES (pH 7.2/NaOH), 0.4 M sucrose, 5 mM MgCl_2_, 15 mM NaCl, 10% glycerol] to a final volume of 30 μL. After a short gentle mix and 2‐min incubation on ice, the samples were centrifuged at 20 000*g*/4°C for 10 min to remove non‐solubilised membranes. The supernatant was loaded onto a polyacrylamide gel with a 4–8% gradient (Wittig et al., 2007) without a stacking gel. The electrophoretic separation was conducted in a Bio‐Rad Mini protean tetra cell system (BioRad, Herkules, USA), starting with a constant current of 3.5 mA for 15 min and then continued with a constant current of 7 mA until the front reached the bottom of the gel. The visualisation of the CN‐PAGE gel was performed using an Amersham Imager 600RGB gel scanner (GE HealthCare Life Sciences, Tokyo, Japan), using both transmission mode with white light illumination for the visualisation of all bands and fluorescence mode with a 460 nm excitation filter and an emission filter of Cy5 (705BP40 = 705 ± 20 nm).

### Mass spectrometry analysis

Selected 1D CN‐PAGE gel bands were excised manually and after washing, each band was subjected to a protein reduction (10 mM DTT in 25 mM NaHCO_3_, 45 min, 56°C, 750 rpm) and an alkylation (55 mM IAA in 25 mM NaHCO_3_; 30 min, room temperature, 750 rpm) step. After further washing by 50% ACN/NaHCO_3_ and pure ACN, the gel pieces were incubated with 125 ng trypsin (Proteomics grade; Merc) in 50 mM NaHCO_3_. The digestion was performed for 2 h at 40°C on a Thermomixer (750 rpm; Eppendorf). Tryptic peptides were extracted into LC–MS vials by 2.5% formic acid (FA) in 50% ACN with the addition of polyethylene glycol (20 000; final concentration 0.001%) (Stejskal et al., 2013) and concentrated in a SpeedVac concentrator (Thermo Fisher Scientific).

LC–MS/MS analyses were done using an UltiMate 3000 RSLCnano system (Thermo Fisher Scientific) connected to either Orbitrap Exploris 480 (Thermo Fisher Scientific) or timsTOF Pro (Bruker) spectrometers. Prior to LC separation, tryptic digests were online concentrated and desalted using a trapping column (Acclaim™ PepMap™ 100 C18, dimensions 300 μm ID, 5 mm long, 5 μm particles, Thermo Fisher Scientific). After washing the trapping column with 0.1% formic acid (FA), the peptides were eluted (flow rate 300 nL min^−1^) from the trapping column onto an analytical column (timsTOF Pro: Aurora C18, 75 μm ID, 250 mm long, 1.6 μm particles, heated to 50°C, Ion Opticks; Exploris 480: EASY‐Spray column, 75 μm ID, 250 mm long, 2 μm particles, Thermo Fisher Scientific) by a 30 min linear gradient programs (3–42% of mobile phase B; mobile phase A: 0.1% FA in water; mobile phase B: 0.1% FA in 80% ACN). Equilibration of the trapping column and the analytical column was done prior to sample injection into the sample loop. In case of timsTOF Pro system, the analytical column was placed inside the Butterfly Heater (Phoenix S&T) and its emitter side was installed inside the CaptiveSpray ion source (Bruker) according to the manufacturer's instructions with the column temperature set to 50°C. In a case of Exploris, the analytical column was installed in the EASY‐Spray ion source (Thermo Fisher Scientific; temperatures set to 50°C) according to the manufacturer's instructions. Spray voltage and sweep gas were set to 1.6 kV and 1, respectively.

MS/MS data on timsTOF Pro were acquired in the m/z range of 100–1700 and the 1/k0 range of 0.6–1.6 V × s × cm^−2^ using the DDA‐PASEF method acquiring 10 PASEF scans with a scheduled target intensity of 20 000 and an intensity threshold of 2500. Active exclusion was set for 0.3 min with precursor reconsideration for 4× more intense precursors. MS/MS data on Exploris were acquired in a data‐dependent strategy (cycle time 1.4 s). The survey scan range was set to *m/z* 350–2000 with the resolution of 60 000 (at *m/z* 200), the normalised target value of 250% and the maximum injection time of 500 ms. HCD MS/MS spectra (isolation window *m/z* 1.2, 30% relative fragmentation energy) were acquired from 110 *m/z* with a relative target value of 200% (intensity threshold 5 × 10^3^), resolution of 30 000 (at *m/z* 200) and maximum injection time of 250 ms. Dynamic exclusion was enabled for 30 s.

For data evaluation, we used the MaxQuant software (v2.4.2.0) (Cox & Mann, 2008) with inbuilt Andromeda search engine (Cox et al., 2011). The search was done against the protein databases of *P. patens* (https://www.uniprot.org/proteomes/UP000006727; version 2023/03, number of protein sequences: 47782) and cRAP contaminants (112 sequences, version from 2018‐11‐22, downloaded from http://www.thegpm.org/crap). Modifications were set as follows for the database search: oxidation (M), deamidation (N, Q), acetylation (Protein N‐term) and carbamidomethylation (C) as variable modifications, with no fixed modification. Enzyme specificity was tryptic/P with two permissible missed cleavages. Only peptides and proteins with a false discovery rate threshold under 0.01 were considered. Match between runs was not enabled. Relative protein abundance was assessed using protein intensities calculated by MaxQuant. Intensities of reported proteins were further evaluated using software container environment (https://github.com/OmicsWorkflows/KNIME_docker_vnc; version 4.7.7a). Processing workflow is available upon request and it covers, in short, reverse hits and contaminant protein groups (cRAP) removal, protein group intensities log2 transformation and normalisation (loessF).

For B1–B3 and B4 band three and four technical replicates were analysed, respectively. The results of MS analysis of band B4 were not used for evaluating relative protein abundance in comparison with bands B1–B3, due to difference in used instrumentation (B1−B3, timsTOF; B4, Exploris).

### Electron microscopy and single particle analysis

Elution of individual CN‐PAGE bands and preparation of specimens for single particle electron microscopy was performed according to a procedure described by Kouřil et al. (2014). Electron micrographs were collected using a Tecnai G2 F20 microscope (FEI Technologies, Hillsboro, USA) with an Eagle 4K CCD camera (FEI Technologies, Hillsboro, USA) at 134 028× magnification. The pixel size at the specimen level after binning the images to 2048 × 2048 pixels was 0.224 nm. Approx. 2478 and 20 454 of projections were picked in semi‐automated mode from 4218 and 5953 micrographs of specimens prepared from the gel bands assigned as B1 and B2, respectively. The two data sets were grouped and subjected to reference‐free 2D classification using the SCIPION image processing framework (de la Rosa‐Trevín et al., 2016). Overall, 15 093 PSI‐NDH and 7839 NDH particles were classified in the first round of classification. The final classification of PSI‐NDH in *Pp* resulted in the identification of two distinct classes. The typical form of PSI‐NDH comprised 2566 particles, while the shifted form included 1098 particles. The final density map of the NDH monomer was obtained by averaging 2725 particles. The obtained electron density maps were fitted with the structure of the NDH complex extracted from the *At* PSI‐NDH sc structure (PDB ID: 7WG5, Su et al., 2022) excluding SubL, PnsB2 and PnsB3 subunits, and the structure of *Pp* PSI (PDB ID: 7KSQ, Gorski et al., 2022).

### 2D‐SDS‐PAGE

2D‐SDS‐PAGE was performed according to Laemmli (1970) with several modifications. The mini gel (*W* × *L* × thickness [cm] = 8.4 × 6.3 × 0.15) was composed of a 5% stacking gel (approx. height 0.5 cm) and a 12% separating gel. The 5% gel was composed of 0.7 M Tris (pH 8.6/HCl), a 10× diluted 50% stock solution of acrylamide (50% T, 2.6% C). The 12% gel was composed of 7 M urea, 0.7 M Tris (pH 8.6/HCl) and a 4.2× diluted 50% stock solution of the acrylamide solution. The cathode (upper) buffer was composed of 25 mM Tris, 0.192 M glycine and 3.5 mM SDS, and the anode (lower) buffer was composed of 25 mM Tris (pH 8.3/HCl).

After incubation of gel strips from the first dimension in SDS buffer (25 mM Tris [pH 7.5/HCl], 35 mM SDS, 15 mM DTT) for 30 min, the strips were applied on the stacking gel and fixed in position by embedding in homogeneously dissolved 0.6% agarose in the upper buffer. The electrophoresis conditions were as follows: 5 mA/30 min =>10 mA/3.83 h at room temperature.

### Western blot analysis on 2D gel

The proteins contained within TM of *Pp* separated in the first dimension by CN‐PAGE and then in the second dimension by SDS‐PAGE were transferred from the gel to a polyvinylidene fluoride membrane using the Trans‐Blot Turbo RTA Mini 0.2 μm PVDF Transfer Kit (BioRad, Herkules, USA) with conditions 2.5 A, 25 V for 30 min. The presence of the NDH complex was detected using a primary Anti‐NdhH antibody (AS16 4065, Agrisera, Vännäs, Sweden) in tandem combination with the secondary antibody with conjugated Horse Radish Peroxidase (HRP) enzyme. The chemiluminescent signal was recorded after developing with Immobilon Western Chemiluminescent HRP Substrate (Merck, Darmstadt, Germany) and visualised using a gel scanner Amersham Imager 600RGB (GE HealthCare Life Sciences, Tokyo, Japan).

## AUTHOR CONTRIBUTIONS

MO planned, designed and performed the experiments and wrote the manuscript. RK supervised the whole research, participated in designing the experiments, analysis of results and correction of the manuscript. Both authors revised and approved the manuscript.

## FUNDING INFORMATION

This work was supported by the Ministry of Education Youth and Sports of the Czech Republic (the Johannes Amos Comenius Programme—Excellent Research, project acronym PHOTOMACHINES, registration number: CZ.02.01.01/00/22_008/0004624).

## CONFLICT OF INTEREST STATEMENT

The authors declare that there is no conflict of interest.

## Supporting information


**Figure S1.** Single particle electron microscopy analysis of the PSI‐NDH supercomplex in *Physcomitrium patens*.
**Figure S2.** Relative content of selected proteins in analysed bands (B1–B3).
**Figure S3.** Impact of PSI rotation within *Pp* PSI‐NDH sc on the distance between the putative ferredoxin (Fd)‐binding sites in NDH and PSI.
**Table S1.** A summary of the identified PSI‐NDH supercomplex subunits in *Physcomitrium patens*.
**Table S2.** The list of all LHCI proteins (including LHCB9) and their isoforms identified in four analysed bands B1, B2, B3 and B4 by mass spectrometry.

## Data Availability

Mass spectrometry proteomics data were deposited to the ProteomeXchange Consortium via the PRIDE (Perez‐Riverol et al., 2022) partner repository under dataset identifier PXD056030 (Reviewer account details: Unique link: https://www.ebi.ac.uk/pride/review‐dataset/6545f191184147c9ba9cfe9da1b43824, Username: reviewer_pxd056030@ebi.ac.uk, Password: OQxoXNjQMOfi). The data that support the findings of this study are openly available in the Zenodo repository (DOI: 10.5281/zenodo.13946196).

## References

[tpj17116-bib-0001] Alboresi, A. , Caffarri, S. , Nogue, F. , Bassi, R. & Morosinotto, T. (2008) *In silico* and biochemical analysis of *Physcomitrella patens* photosynthetic antenna: identification of subunits which evolved upon land adaptation. PLoS One, 3, e2033. Available from: 10.1371/journal.pone.0002033 18446222 PMC2323573

[tpj17116-bib-0002] Allen, J.F. (2002) Photosynthesis of ATP‐electrons, proton pumps, rotors, and poise. Cell, 110, 273–276. Available from: 10.1016/S0092-8674(02)00870-X 12176312

[tpj17116-bib-0003] Amunts, A. , Toporik, H. , Borovikova, A. & Nelson, N. (2010) Structure determination and improved model of plant photosystem I. The Journal of Biological Chemistry, 285, 3478–3486. Available from: 10.1074/jbc.M109.072645 19923216 PMC2823434

[tpj17116-bib-0004] Armbruster, U. , Rühle, T. , Kreller, R. , Strotbek, C. , Zühlke, J. , Tadini, L. et al. (2013) The photosynthesis affected mutant68‐like protein evolved from a PSII assembly factor to mediate assembly of the chloroplast NAD(P)H dehydrogenase complex in Arabidopsis. Plant Cell, 25, 3926–3943. Available from: 10.1105/tpc.113.114785 24096342 PMC3877787

[tpj17116-bib-0005] Basso, L. , Yamori, W. , Szabo, I. & Shikanai, T. (2020) Collaboration between NDH and KEA3 allows maximally efficient photosynthesis after a long dark adaptation. Plant Physiology, 184, 2078–2090. Available from: 10.1104/pp.20.01069 32978277 PMC7723091

[tpj17116-bib-0006] Braukmann, T.W.A. , Kuzmina, M. & Stefanovic, S. (2009) Loss of all plastid *ndh* genes in Gnetales and conifers: extent and evolutionary significance for the seed plant phylogeny. Current Genetics, 55, 323–337. Available from: 10.1007/s00294-009-0249-7 19449185

[tpj17116-bib-0007] Busch, A. , Petersen, J. , Webber‐Birungi, M.T. , Powikrowska, M. , Lassen, L.M. et al. (2013) Composition and structure of photosystem I in the moss *Physcomitrella patens* . Journal of Experimental Botany, 64, 2689–2699. Available from: 10.1093/jxb/ert126 23682117 PMC3697952

[tpj17116-bib-0008] Cox, J. & Mann, M. (2008) MaxQuant enables high peptide identification rates, individualized p.p.b.‐range mass accuracies and proteome‐wide protein quantification. Nature Biotechnology, 26, 1367–1372. Available from: 10.1038/nbt.1511 19029910

[tpj17116-bib-0009] Cox, J. , Neuhauser, N. , Michalski, A. , Scheltema, R.A. , Olsen, J.V. & Mann, M. (2011) Andromeda: a peptide search engine integrated into the MaxQuant environment. Journal of Proteome Research, 10, 1794–1805. Available from: 10.1021/pr101065j 21254760

[tpj17116-bib-0010] DalCorso, G. , Pesaresi, P. , Masiero, S. , Aseeva, E. , Schünemann, D. , Finazzi, G. et al. (2008) A complex containing PGRL1 and PGR5 is involved in the switch between linear and cyclic electron flow in Arabidopsis. Cell, 132, 273–285. Available from: 10.1016/j.cell.2007.12.028 18243102

[tpj17116-bib-0011] Dau, H. , Andrews, J.C. , Roelofs, T.A. , Latimer, M.J. , Liang, W. , Yachandra, V.K. et al. (1995) Structural consequences of ammonia binding to the manganese center of the photosynthetic oxygen‐evolving complex: an X‐ray absorption spectroscopy study of isotropic and oriented photosystem II particles. Biochemistry, 34, 5274–5287. Available from: 10.1021/bi00015a043 7711049

[tpj17116-bib-0012] de la Rosa‐Trevín, J.M. , Quintana, A. , Del Cano, L. , Zaldívar, A. , Foche, I. et al. (2016) Scipion: a software framework toward integration, reproducibility and validation in 3D electron microscopy. Journal of Structural Biology, 195, 93–99. Available from: 10.1016/j.jsb.2016.04.010 27108186

[tpj17116-bib-0013] Gao, F. , Zhao, J. , Chen, L. , Battchikova, N. , Ran, Z. , Aro, E.M. et al. (2016) The NDH‐1 L‐PSI supercomplex is important for efficient cyclic electron transport in cyanobacteria. Plant Physiology, 172, 1451–1464. Available from: 10.1104/pp.16.00585 27621424 PMC5100770

[tpj17116-bib-0014] Gorski, C. , Riddle, R. , Toporik, H. , Da, Z. , Dobson, Z. , Williams, D. et al. (2022) The structure of the *Physcomitrium patens* photosystem I reveals a unique Lhca2 paralogue replacing Lhca4. Nature Plants, 8, 307–316. Available from: 10.1038/s41477-022-01099-w 35190662

[tpj17116-bib-0015] Grebe, S. , Trotta, A. , Bajwa, A.A. , Suorsa, M. , Gollan, P.J. , Jansson, S. et al. (2019) The unique photosynthetic apparatus of Pinaceae: analysis of photosynthetic complexes in *Picea abies* . Journal of Experimental Botany, 70, 3211–3225. Available from: 10.1093/jxb/erz127 30938447 PMC6598058

[tpj17116-bib-0016] Hertle, A.P. , Blunder, T. , Wunder, T. , Pesaresi, P. , Pribil, M. , Armbruster, U. et al. (2013) PGRL1 is the elusive ferredoxin‐plastoquinone reductase in photosynthetic cyclic electron flow. Molecular Cell, 49, 511–523. Available from: 10.1016/j.molcel.2012.11.030 23290914

[tpj17116-bib-0017] Ifuku, K. , Endo, T. , Shikanai, T. & Aro, E.M. (2011) Structure of the chloroplast NADH dehydrogenase‐like complex: nomenclature for nuclear‐encoded subunits. Plant & Cell Physiology, 52, 1560–1568. Available from: 10.1093/pcp/pcr098 21785130

[tpj17116-bib-0018] Iwai, M. , Grob, P. , Iavarone, A.T. , Nogales, E. & Niyogi, K.K. (2018) A unique supramolecular organization of photosystem I in the moss *Physcomitrella patens* . Nature Plants, 4, 904–909. Available from: 10.1038/s41477-018-0271-1 30374090 PMC7806276

[tpj17116-bib-0019] Iwai, M. & Yokono, M. (2017) Light‐harvesting antenna complexes in the moss *Physcomitrella patens*: implications for the evolutionary transition from green algae to land plants. Current Opinion in Plant Biology, 37, 94–101. Available from: 10.1016/j.pbi.2017.04.002 28445834

[tpj17116-bib-0020] Iwai, M. , Yokono, M. , Kono, M. , Noguchi, K. , Akimoto, S. & Nakano, A. (2015) Light‐harvesting complex Lhcb9 confers a green alga‐type photosystem I supercomplex to the moss *Physcomitrella patens* . Nature Plants, 1, 14008. Available from: 10.1038/nplants.2014.8 27246756

[tpj17116-bib-0021] Kato, Y. , Odahara, M. , Fukao, Y. & Shikanai, T. (2018) Stepwise evolution of supercomplex formation with photosystem I is required for stabilization of chloroplast NADH dehydrogenase‐like complex: Lhca5‐dependent supercomplex formation in *Physcomitrella patens* . The Plant Journal, 96, 937–948. Available from: 10.1111/tpj.14080 30176081

[tpj17116-bib-0022] Kato, Y. , Odahara, M. & Shikanai, T. (2021) Evolution of an assembly factor‐based subunit contributed to a novel NDH‐PSI supercomplex formation in chloroplasts. Nature Communications, 12, 3685. Available from: 10.1038/s41467-021-24065-0 PMC8211685 34140516

[tpj17116-bib-0023] Kato, Y. , Sugimoto, K. & Shikanai, T. (2018) NDH‐PSI supercomplex assembly precedes full assembly of the NDH complex in chloroplast. Plant Physiology, 176, 1728–1738. Available from: 10.1104/pp.17.01120 29203556 PMC5813578

[tpj17116-bib-0024] Kono, M. & Terashima, I. (2016) Elucidation of photoprotective mechanisms of PSI against fluctuating light photoinhibition. Plant & Cell Physiology, 57, 1405–1414. Available from: 10.1093/pcp/pcw103 27354420

[tpj17116-bib-0025] Kouřil, R. , Strouhal, O. , Nosek, L. , Lenobel, R. , Chamrád, I. , Boekema, E.J. et al. (2014) Structural characterization of a plant photosystem I and NAD(P)H dehydrogenase supercomplex. The Plant Journal, 77, 568–576. Available from: 10.1111/tpj.12402 24313886

[tpj17116-bib-0026] Laemmli, U.K. (1970) Cleavage of structural proteins during the assembly of the head of bacteriophage T4. Nature, 227, 680–685. Available from: 10.1038/227680a0 5432063

[tpj17116-bib-0027] Laughlin, T.G. , Bayne, A.N. , Trempe, J.F. , Savage, D.F. & Davies, K.M. (2019) Structure of the complex I‐like molecule NDH of oxygenic photosynthesis. Nature, 566, 411–414. Available from: 10.1038/s41586-019-0921-0 30742075

[tpj17116-bib-0028] Lichtenthaler, H.K. (1987) Chlorophylls and carotenoids: pigments of photosynthetic biomembranes. Methods in Enzymology, 148, 350–382. Available from: 10.1016/0076-6879(87)48036-1

[tpj17116-bib-0029] Mazor, Y. , Borovikova, A. & Nelson, N. (2015) The structure of plant photosystem I super‐complex at 2.8 Å resolution. eLife, 4, e07433. Available from: 10.7554/eLife.07433 26076232 PMC4487076

[tpj17116-bib-0030] Munekage, Y. , Hojo, M. , Meurer, J. , Endo, T. , Tasaka, M. & Shikanai, T. (2002) PGR5 is involved in cyclic electron flow around photosystem I and is essential for photoprotection in Arabidopsis. Cell, 110, 361–371. Available from: 10.1016/S0092-8674(02)00867-X 12176323

[tpj17116-bib-0031] Niu, Y. , Matsubara, S. , Nedbal, L. & Lazár, D. (2024) Dynamics and interplay of photosynthetic regulatory processes depend on the amplitudes of oscillating light. Plant, Cell & Environment, 47, 2240–2257. Available from: 10.1111/pce.14879 38482712

[tpj17116-bib-0032] Nosek, L. , Semchonok, D. , Boekema, E.J. , Ilík, P. & Kouřil, R. (2017) Structural variability of plant photosystem II megacomplexes in thylakoid membranes. The Plant Journal, 89, 104–111. Available from: 10.1111/tpj.13325 27598242

[tpj17116-bib-0033] Nystedt, B. , Street, N.R. , Wetterbom, A. , Zuccolo, A. , Lin, Y.C. , Scofield, D.G. et al. (2013) The Norway spruce genome sequence and conifer genome evolution. Nature, 497, 579–584. Available from: 10.1038/nature12211 23698360

[tpj17116-bib-0034] Otani, T. , Kato, Y. & Shikanai, T. (2018) Specific substitutions of light‐harvesting complex I proteins associated with photosystem I are required for supercomplex formation with chloroplast NADH dehydrogenase‐like complex. The Plant Journal, 94, 122–130. Available from: 10.1111/tpj.13846 29385648

[tpj17116-bib-0035] Pan, X. , Cao, D. , Xie, F. , Xu, F. , Su, X. , Mi, H. et al. (2020) Structural basis for electron transport mechanism of complex I‐like photosynthetic NAD(P)H dehydrogenase. Nature Communications, 11, 610. Available from: 10.1038/s41467-020-14456-0 PMC6992706 32001694

[tpj17116-bib-0036] Peng, L. , Fukao, Y. , Fujiwara, M. & Shikanai, T. (2012) Multistep assembly of chloroplast NADH dehydrogenase‐like subcomplex a requires several nucleus‐encoded proteins, including CRR41 and CRR42, in Arabidopsis. The Plant Cell, 24, 202–214. Available from: 10.1105/tpc.111.090597 22274627 PMC3289569

[tpj17116-bib-0037] Peng, L. , Fukao, Y. , Fujiwara, M. , Takami, T. & Shikanai, T. (2009) Efficient operation of NAD(P)H dehydrogenase requires supercomplex formation with photosystem I via minor LHCI in Arabidopsis. The Plant Cell, 21, 3623–3640. Available from: 10.1105/tpc.109.068791 19903870 PMC2798312

[tpj17116-bib-0038] Peng, L. & Shikanai, T. (2011) Supercomplex formation with photosystem I is required for the stabilization of the chloroplast NADH dehydrogenase‐like complex in Arabidopsis. Plant Physiology, 155, 1629–1639. Available from: 10.1104/pp.110.171264 21278308 PMC3091109

[tpj17116-bib-0039] Perez‐Riverol, Y. , Bai, J. , Bandla, C. , Hewapathirana, S. , García‐Seisdedos, D. , Kamatchinathan, S. et al. (2022) The PRIDE database resources in 2022: a hub for mass spectrometry‐based proteomics evidences. Nucleic Acids Research, 50, D543–D552. Available from: 10.1093/nar/gkab1038 34723319 PMC8728295

[tpj17116-bib-0040] Pinnola, A. , Alboresi, A. , Nosek, L. , Semchonok, D. , Rameez, A. , Trotta, A. et al. (2018) A LHCB9‐dependent photosystem I megacomplex induced under low light in *Physcomitrella patens* . Nature Plants, 4, 910–919. Available from: 10.1038/s41477-018-0270-2 30374091

[tpj17116-bib-0041] Porada, P. , Lenton, T. , Pohl, A. , Weber, B. , Mander, L. , Donnadieu, Y. et al. (2016) High potential for weathering and climate effects of non‐vascular vegetation in the Late Ordovician. Nature Communications, 7, 12113. Available from: 10.1038/ncomms12113 PMC4941054 27385026

[tpj17116-bib-0042] Qin, X. , Pi, X. , Wang, W. , Han, G. , Zhu, L. , Liu, M. et al. (2019) Structure of a green algal photosystem I in complex with a large number of light‐harvesting complex I subunits. Nature Plants, 5, 263–272. Available from: 10.1038/s41477-019-0379-y 30850820

[tpj17116-bib-0043] Rensing, S.A. , Lang, D. , Zimmer, A.D. , Terry, A. , Salamov, A. , Shapiro, H. et al. (2008) The Physcomitrella genome reveals evolutionary insights into the conquest of land by plants. Science, 319, 64–69. Available from: 10.1126/science.1150646 18079367

[tpj17116-bib-0044] Reski, R. & Abel, W.O. (1985) Induction of budding on chloronemata and caulonemata of the moss, *Physcomitrella patens*, using isopentenyladenine. Planta, 165, 354–358. 24241140 10.1007/BF00392232

[tpj17116-bib-0045] Schuller, J.M. , Birrell, J.A. , Tanaka, H. , Konuma, T. , Wulfhorst, H. , Cox, N. et al. (2019) Structural adaptations of photosynthetic complex I enable ferredoxin‐dependent electron transfer. Science, 363, 257–260. Available from: 10.1126/science.aau3613 30573545

[tpj17116-bib-0046] Shaw, A.J. , Szövényi, P. & Shaw, B. (2011) Bryophyte diversity and evolution: windows into the early evolution of land plants. American Journal of Botany, 98, 352–369. Available from: 10.3732/ajb.1000316 21613131

[tpj17116-bib-0047] Shen, L. , Tang, K. , Wang, W. , Wang, C. , Wu, H. , Mao, Z. et al. (2022) Architecture of the chloroplast PSI‐NDH supercomplex in *Hordeum vulgare* . Nature, 601, 649–654. Available from: 10.1038/s41586-021-04277-6 34879391

[tpj17116-bib-0048] Shikanai, T. (2016) Chloroplast NDH: a different enzyme with a structure similar to that of respiratory NADH dehydrogenase. Biochimica et Biophysica Acta (BBA) ‐ Bioenergetics, 1857, 1015–1022. Available from: 10.1016/j.bbabio.2015.10.013 26519774

[tpj17116-bib-0049] Shikanai, T. (2020) Chapter 6—Regulation of photosynthesis by cyclic electron transport around photosystem I. In: Hisabori, T. (Ed.) Advances in botanical research, Vol. 96. Cambridge: Academic Press, pp. 177–204. Available from: 10.1016/bs.abr.2020.07.005

[tpj17116-bib-0050] Stejskal, K. , Potěšil, D. & Zdráhal, Z. (2013) Suppression of peptide sample losses in autosampler vials. Journal of Proteome Research, 12, 3057–3062. Available from: 10.1021/pr400183v 23590590

[tpj17116-bib-0051] Storti, M. , Puggioni, M.P. , Segalla, A. , Morosinotto, T. & Alboresi, A. (2020) The chloroplast NADH dehydrogenase‐like complex influences the photosynthetic activity of the moss *Physcomitrella patens* . Journal of Experimental Botany, 71, 5538–5548. Available from: 10.1093/jxb/eraa274 32497206

[tpj17116-bib-0052] Su, X. , Cao, D. , Pan, X. , Shi, L. , Liu, Z. , Dall'Osto, L. et al. (2022) Supramolecular assembly of chloroplast NADH dehydrogenase‐like complex with photosystem I from *Arabidopsis thaliana* . Molecular Plant, 15, 454–467. Available from: 10.1016/j.molp.2022.01.020 35123031

[tpj17116-bib-0053] Suga, M. , Ozawa, S.I. , Yoshida‐Motomura, K. , Akita, F. , Miyazaki, N. & Takahashi, Y. (2019) Structure of the green algal photosystem I supercomplex with a decameric light‐harvesting complex I. Nature Plants, 5, 626–636. Available from: 10.1038/s41477-019-0438-4 31182847

[tpj17116-bib-0054] Sugimoto, K. , Okegawa, Y. , Tohri, A. , Long, T.A. , Covert, S.F. , Hisabori, T. et al. (2013) A single amino acid alteration in PGR5 confers resistance to antimycin A in cyclic electron transport around PSI. Plant & Cell Physiology, 54, 1525–1534. Available from: 10.1093/pcp/pct098 23872270

[tpj17116-bib-0055] Sun, H. , Shang, H. , Pan, X. & Li, M. (2023) Structural insights into the assembly and energy transfer of the Lhcb9‐dependent photosystem I from moss *Physcomitrium patens* . Nature Plants, 9, 1347–1358. Available from: 10.1038/s41477-023-01463-4 37474782

[tpj17116-bib-0056] Tagawa, K. , Tsujimoto, H.Y. & Arnon, D.I. (1963) Role of chloroplast ferredoxin in the energy conversion process of photosynthesis. Proceedings of the National Academy of Sciences of the United States of America, 49, 567–752. Available from: 10.1073/pnas.49.4.567 13980171 PMC299906

[tpj17116-bib-0057] Ueda, M. , Kuniyoshi, T. , Yamamoto, H. , Sugimoto, K. , Ishizaki, K. , Kohchi, T. et al. (2012) Composition and physiological function of the chloroplast NADH dehydrogenase‐like complex in *Marchantia polymorpha* . The Plant Journal, 72, 683–693. Available from: 10.1111/j.1365-313X.2012.05115.x 22862786

[tpj17116-bib-0058] Wittig, I. , Karas, M. & Schägger, H. (2007) High resolution clear native electrophoresis for in‐gel functional assays and fluorescence studies of membrane protein complexes. Molecular & Cellular Proteomics, 6, 1215–1225. Available from: 10.1074/mcp.M700076-MCP200 17426019

[tpj17116-bib-0059] Yamamoto, H. , Fan, X. , Sugimoto, K. , Fukao, Y. , Peng, L. & Shikanai, T. (2016) CHLORORESPIRATORY REDUCTION 9 is a novel factor required for formation of subcomplex A of the chloroplast NADH dehydrogenase‐like complex. Plant & Cell Physiology, 57, 2122–2132. Available from: 10.1093/pcp/pcw130 27481895

[tpj17116-bib-0060] Yamamoto, H. , Sato, N. & Shikanai, T. (2021) Critical role of NdhA in the incorporation of the peripheral arm into the membrane‐embedded part of the chloroplast NADH dehydrogenase‐like complex. Plant & Cell Physiology, 62, 1131–1145. Available from: 10.1093/pcp/pcaa143 33169158

[tpj17116-bib-0061] Yamori, W. , Makino, A. & Shikanai, T. (2016) A physiological role of cyclic electron transport around photosystem I in sustaining photosynthesis under fluctuating light in rice. Scientific Reports, 6, 20147. Available from: 10.1038/srep20147 26832990 PMC4735858

[tpj17116-bib-0062] Yamori, W. , Sakata, N. , Suzuki, Y. , Shikanai, T. & Makino, A. (2011) Cyclic electron flow around photosystem I via chloroplast NAD(P)H dehydrogenase (NDH) complex performs a significant physiological role during photosynthesis and plant growth at low temperature in rice. The Plant Journal, 68, 966–976. Available from: 10.1111/j.1365-313X.2011.04747.x 21848656

[tpj17116-bib-0063] Yamori, W. , Shikanai, T. & Makino, A. (2015) Photosystem I cyclic electron flow via chloroplast NADH dehydrogenase‐like complex performs a physiological role for photosynthesis at low light. Scientific Reports, 5, 13908. Available from: 10.1038/srep13908 26358849 PMC4566099

[tpj17116-bib-0064] Yan, Q. , Zhao, L. , Wang, W. , Pi, X. , Han, G. , Wang, J. et al. (2021) Antenna arrangement and energy‐transfer pathways of PSI‐LHCI from the moss *Physcomitrella patens* . Cell Discovery, 7, 10. Available from: 10.1038/s41421-021-00242-9 33589616 PMC7884438

[tpj17116-bib-0065] Zhang, S. , Tang, K. , Yan, Q. , Li, X. , Shen, L. , Wang, W. et al. (2023) Structural insights into a unique PSI–LHCI–LHCII–Lhcb9 supercomplex from moss *Physcomitrium patens* . Nature Plants, 9, 832–846. Available from: 10.1038/s41477-023-01401-4 37095225

[tpj17116-bib-0066] Zhang, Z. , Zhao, L.‐S. & Liu, L.‐N. (2021) Characterizing the supercomplex association of photosynthetic complexes in cyanobacteria. Royal Society Open Science, 8, 202142. Available from: 10.1098/rsos.202142 34295515 PMC8278045

[tpj17116-bib-0067] Zimmer, A.D. , Lang, D. , Buchta, K. , Rombauts, S. , Nishiyama, T. , Hasebe, M. et al. (2013) Reannotation and extended community resources for the genome of the non‐seed plant *Physcomitrella patens* provide insights into the evolution of plant gene structures and functions. BMC Genomics, 14, 498. Available from: 10.1186/1471-2164-14-498 23879659 PMC3729371

