# Supplementary material for: Unique structural attributes of the PSI‐NDH supercomplex in Physcomitrium patens
Source: Plant J. 2024 Nov 3;120(5):2226–37. doi: 10.1111/tpj.17116 (PMC11629750; doi:10.1111/tpj.17116)
Supplement: Supplementary file 1 — Figure S1. Single particle electron microscopy analysis of the PSI‐NDH supercomplex in Physcomitrium patens. Figure S2. Relative content of selected proteins in analysed bands (B1–B3). Figure S3. Impact of PSI rotation within Pp PSI‐NDH sc on the distance between the putative ferredoxin (Fd)‐binding sites in NDH and PSI. Table S1. A summary of the identified PSI‐NDH supercomplex subunits in Physcomitrium patens. Table S2. The list of all LHCI proteins (including LHCB9) and their isoforms identified in four analysed bands B1, B2, B3 and B4 by mass spectrometry. [file TPJ-120-2226-s001.docx]

**Supporting information**


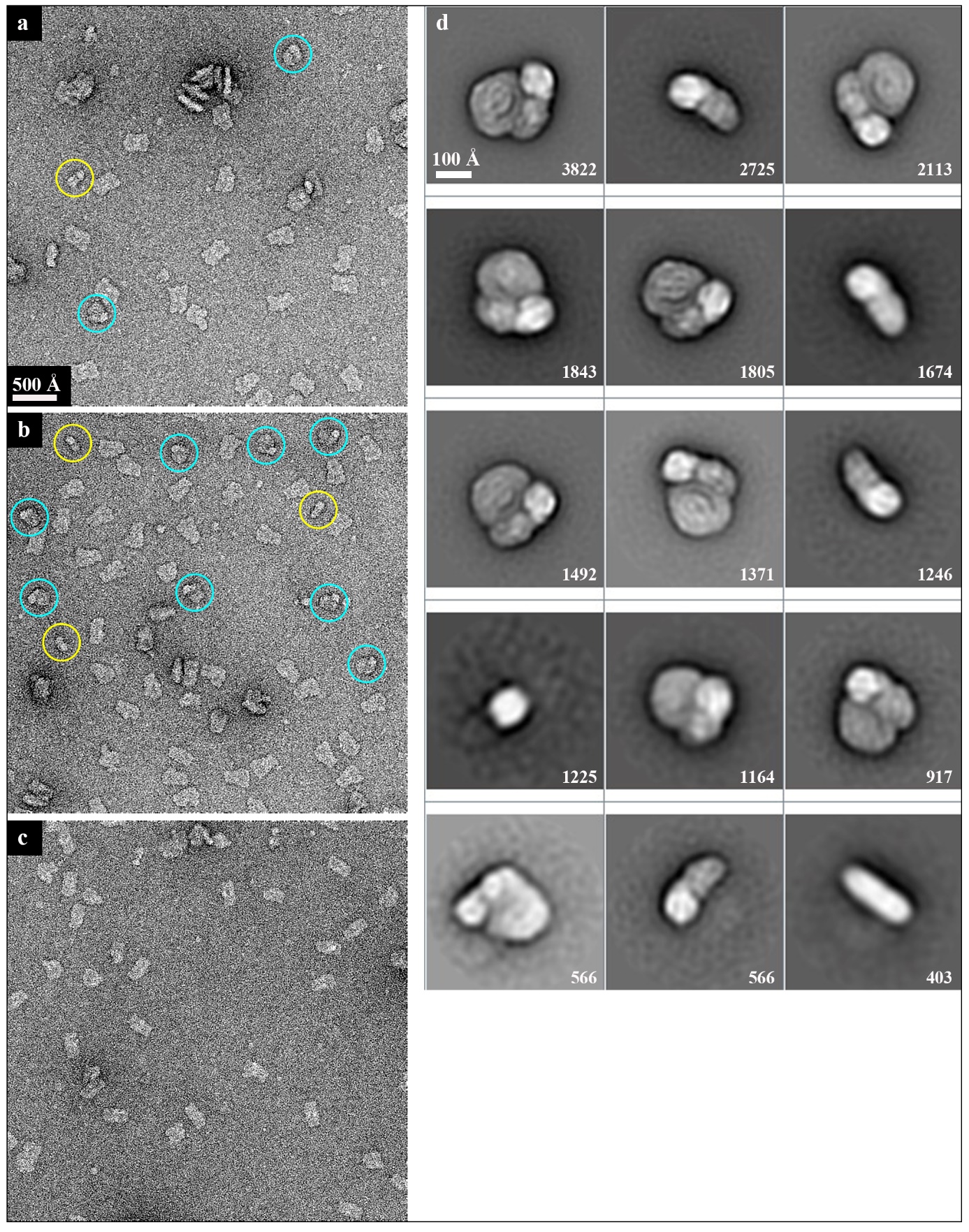


**Supporting Figure S1: Single particle electron microscopy analysis of PSI-NDH supercomplex in *Physcomitrium patens*.** Representative transmission EM micrographs of the **a)** B1, **b)** B2 and **c)** B3 band containing PSII scs (un-circled particles), PSI-NDH scs (in blue circles) and NDH monomers (in yellow circles); **d)** 2D classification of the PSI-NDH and NDH particles extracted from all micrographs of B1 and B2 bands with the given numbers of particles in each class. The 2478 and 20 454 particles were extracted for B1 and B2 band, respectively, and were further classified into 15 classes.


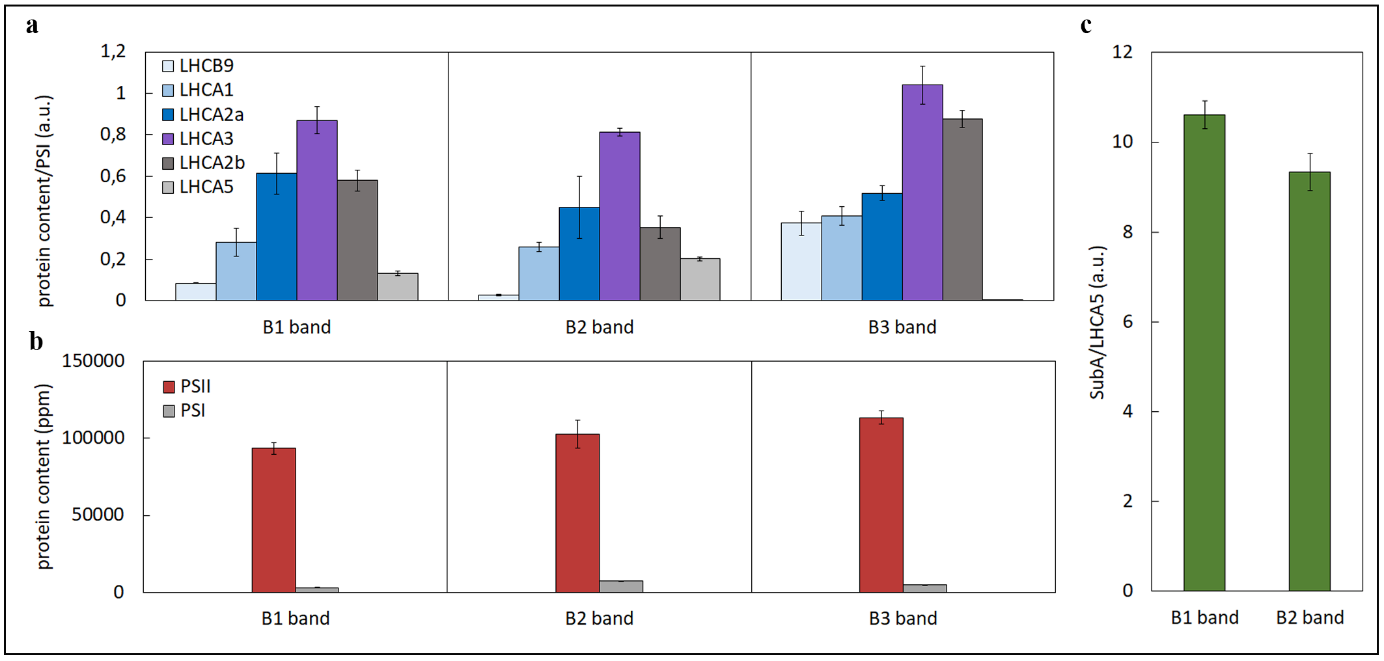


**Supporting Figure S2:** **Relative content of selected proteins in analysed bands (B1–B3). a)** Relative protein abundance of individual LHCI proteins (including LHCB9) evaluated to the protein content of PSI core (represented by the sum of PsaA and PsaB) in three different bands: B1, B2, and B3. The relative protein content of all isoforms representing one LHCA protein (see Table S2) were summarised and subsequently evaluated as one for reducing results complicity. **b)** The relative protein content of PSI (represented by sum of PsaA and PsaB) and PSII (represented by sum of PsbA(D1) and PsbD (D2)) evaluated to the sum of all proteins in specific bands. **c)** The SubA/PSI ratio calculated as the sum of relative protein abundance of SubA NDH subunits divided by the sum of relative protein content of PsaA and PsaB in bands B1 and B2. The relative content (relative PG iBAQ values (riBAQ) in ppm format) of individual LHCA isoforms, LHCB9, SubA subunits, PsaA, PsaB, PsbA (D1) and PsbD (D2) was determined by mass spectrometry in samples prepared from gel bands excised from CN-PAGE (Fig 1a). The presented values are means ± SD from 3 replicates.


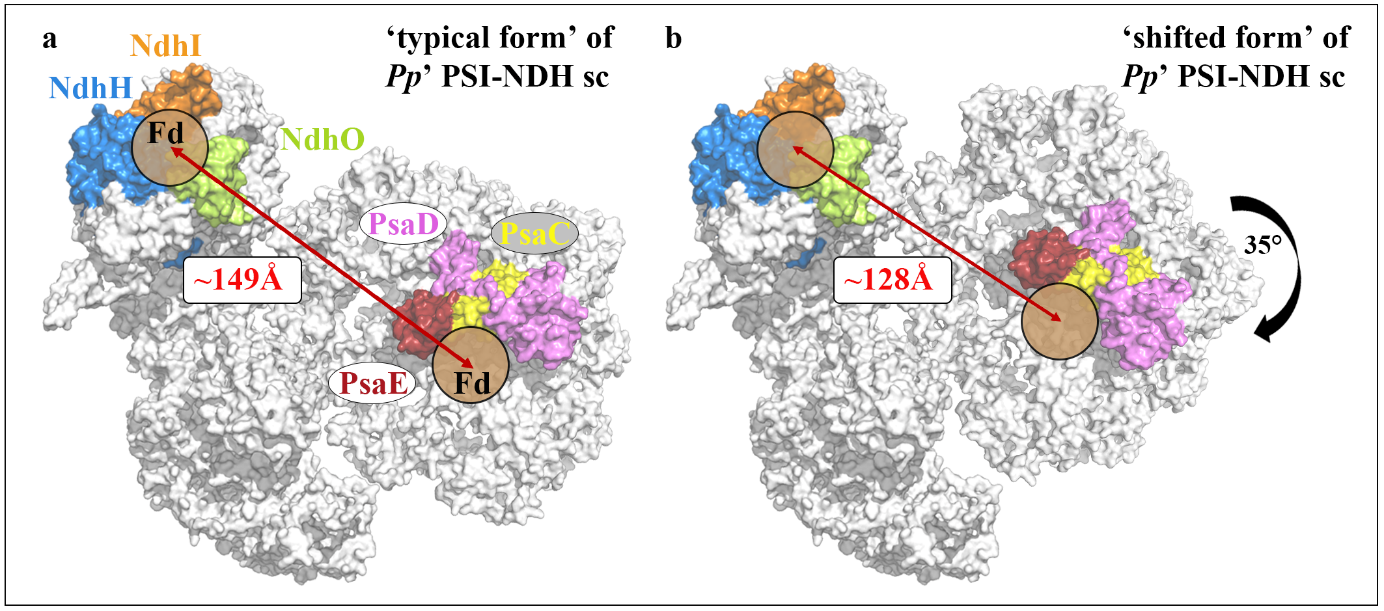
**Supporting Figure S3: Impact of PSI rotation within Pp PSI-NDH sc on the distance between the putative ferredoxin (Fd)-binding sites in NDH and PSI.** Comparison of the distance between Fd-binding site in NDH and PSI in typical **(a)** and shifted **(b)** form of PSI-NDH sc. The subunits forming Fd-binding site in NDH according Laughlin et al. (2020): NdhO, NdhI, NdhH (excluding NdhS and NdhV absent in model) and in PSI according Caspy et al. (2020): stromal PsaC-E (excluding PsaA, PsaF) are labelled and colour coded as follows: NDH: limon, blue, orange – NdhO, NdhH, NdhI, respectively; PSI: yellow, violet, and firebrick –PsaC, PsaD, PsaE, respectively, with the rest of the subunits in PSI and NDH in grey colour. The binding positions of the Fd in NDH (O-site) and PSI are illustrated as brown circles. Structural models of PSI-NDH scs viewed from the stromal side were obtained by a fit of the NDH monomer from At (PDB ID: 7WG5, Su et al., 2022) and PSI complex from Pp (PDB ID: 7KSQ, Gorski et al., 2022). Prior to fitting, the subunits of NDH unencoded in Pp genome were removed from the At NDH structure, namely the PnsB2, PnsB3, and the subunits of SubL. Two headed arrows determine the distance between approx. centre of Fd-binding sites in NDH and PSI in two different structures with estimated values in Angstroms.

**Supporting Table S1:** **A summary of the identified PSI-NDH supercomplex subunits in Physcomitrium patens.** MS analysis of the B2 band (in 3 technical replicates) from CN-PAGE used for transmission EM analysis. The subunits of the PSI and NDH complexes identified in the band after digestion with trypsin and assigned according to UniProt database records are listed. Proteins highlighted in green are not encoded in the Physcomitrium patens genome or do not have any record in the UniProt database, proteins highlighted in orange were not identified in our dataset.

|  |  | **Protein** | **Molecular weight (kDa)** | **UniProt accession number** | **Peptides (all/unique)** | **Sequence coverage (%)** |
| --- | --- | --- | --- | --- | --- | --- |
| **NDH** | **SubM** | NdhA | 41,1 | Q6YXP8 | 8/8 | 26.1 |
|  |  | NdhB | 55,9 | Q6YXR9 | 2/2 | 9.8 |
|  |  | NdhC | 14 | Q6YXR0 | 1/1 | 9.9 |
|  |  | NdhD | 56,5 | Q6YXQ3 | 4/4 | 9.4 |
|  |  | NdhE | 11,1 | Q6YXQ1 | 2/2 | 9.0 |
|  |  | NdhF | 81,9 | Q6YXQ6 | 12/12 | 15.7 |
|  |  | NdhG | 22,2 | Q6YXQ0 | 5/5 | 16.5 |
|  | **SubA** | NdhH | 45,3 | Q6YXP7 | 31/31 | 72.4 |
|  |  | NdhI | 20,9 | Q6YXP9 | 13/13 | 66.9 |
|  |  | NdhJ | 19,9 | Q6YXQ8 | 15/15 | 81.1 |
|  |  | NdhK | 29,6 | Q6YXQ9 | 25/25 | 86.2 |
|  |  | NdhL | 21,8 | A0A2K1IH93 | 3/3 | 9.7 |
|  |  | NdhM | 23,1 | A9THA7 | 13/13 | 58.8 |
|  |  | NdhN | 27,6 | A0A2K1KUL0 | 12/12 | 49.0 |
|  |  | NdhO | 19,2 | A9TH99 | 1/1 | 5.7 |
|  | **SubB** | PnsB1 | 51,5 | A9SCR2 | 230/26 | 69.9 |
|  |  | PnsB2–B3 |  | *Unencoded* |  |  |
|  |  | PnsB4 | 23,3 | A0A2K1J732 | 6/6 | 25.7 |
|  |  | PnsB5 | 13,6 | A0A7I4C6K0 | 4/4 | 39.1 |
|  | **SubE** | NdhS | 30,8 | A0A2K1JVE5 | 18/18 | 56.8 |
|  |  | NdhT | 27,8 | A0A2K1J830 | 7/7 | 35.9 |
|  |  | NdhU | 25,3 | A9RK51 | 14/7 | 56.3 |
|  |  | NdhV | 22.4 | *No record* |  |  |
|  | **SubL** | PnsL1–L5 |  | *Unencoded* |  |  |
| **PSI-LHCI** | **PSI** | PsaA | 83,1 | Q8MFA3 | 16/15 | 19.3 |
|  |  | PsaB | 82,4 | Q8MFA2 | 11/10 | 18.5 |
|  |  | PsaC | 8,8 | Q6YXQ2 | 7/7 | 85.2 |
|  |  | PsaD | 19,9 | A0A7I4B8M6 | 15/3 | 77.0 |
|  |  | PsaE | 13,9 | A0A2K1IHL7 | 9/3 | 47.7 |
|  |  | PsaF | 26,1 | A0A2K1IN36 | 6/2 | 21.1 |
|  |  | PsaG | 16,2 | A0A2K1JC42 | 3/2 | 22.6 |
|  |  | PsaH | 14,5 | A0A2K1JDR4 | 6/2 | 55.4 |
|  |  | PsaI | 4 | *Not identified* | - | - |
|  |  | PsaJ | 4,6 | *Not identified* | - | - |
|  |  | PsaK | 12 | A0A2K1KU02 | 3/3 | 34.8 |
|  |  | PsaL | 23,5 | A0A2K1IAD0 | 4/2 | 20.5 |
|  |  | PsaM | 3,4 | *Not identified* | - | - |
|  |  | PsaN |  | *Unencoded* |  |  |
|  |  | PsaO | 15,4/16,4 | *Not identified* | - | - |
|  | **LHCI** | LHCA1 | 25,9 | A0A2K1JLZ3 | 8/3 | 39.1 |
|  |  |  |  | A0A2K1KU97 | 9/5 | 43.4 |
|  |  |  |  | A0A2K1KQE9 | 8/3 | 43 |
|  |  | LHCA2a | 29 | A9TJ06 | 5/3 | 27.8 |
|  |  |  |  | A0A2K1K0C7 | 6/2 | 36.6 |
|  |  |  |  | A0A2K1JRL9 | 5/1 | 27.9 |
|  |  | LHCA2b | 28,8 | A0A2K1K0E4 | 11/11 | 51.9 |
|  |  | LHCA3 | 30 | A0A2K1IB07 | 8/0 | 24.4 |
|  |  |  |  |  |  |  |
|  |  |  |  | A0A2K1J8T2 | 6/1 | 22.3 |
|  |  | LHCA5 | 29,6 | A0A2K1KN29 | 4/4 | 22.3 |

**Supporting Table S2: The list of all LHCI proteins (including LHCB9) and their isoforms identified in four analysed bands B1, B2, B3 and B4 by mass spectrometry.** Individual rows represent different isoforms of LHCI antennae detected in specific bands by MS, accession numbers according UniProt database in one row represent the same isoform of protein. Letters “Y” and “N” indicate whether the specific protein was detected or undetected in MS dataset of individual bands, respectively. The label “Low” was assigned to those protein that were detected, however, whose abundance was close to the detection limit or they had overall low and negligible abundance relatively to PSI core. For band B1–B3 and B4 three and four technical replicates were analysed, respectively.

| **LHCI protein** | **UniProt accession number** | **B1 band** | **B2 band** | **B3 band** | **B4 band** |
| --- | --- | --- | --- | --- | --- |
| **LHCA1** | A0A2K1JLZ3 | Y | Y | Y | Y |
|  | A0A2K1KU97/A0A7I4D7K2 | Y | Y | Y | Y |
|  | A0A2K1KQE9/A0A7I4DLH8/A0A7I4DUB8 | Y | Y | Y | Y |
| **LHCA2a** | A9TJ06/A0A2K1IW94 | Y | Y | Y | Y |
|  | A0A2K1K0C7 | Y | Y | Y | Y |
|  | A0A2K1JRL9/A0A7I4ACT6/A9U5E4 | Y *(Low)* | Y *(Low)* | Y *(Low)* | **N** |
| **LHCA2b** | A0A2K1K0E4/A0A2K1K0D5/A0A7I4A1C5 | Y | Y | Y | Y |
| **LHCA3** | A0A2K1IB07/A0A2K1IB10 | Y | Y | Y | Y |
|  | A0A2K1J8T2/A0A7I4F6K9 | Y | Y | Y | Y |
| **LHCA5** | A0A2K1KN29 | Y | Y | Y *(Low)* | Y *(Low)* |
| **LHCB9** | A0A2K1KER4 | Y | Y | Y | **N** |
|  | A0A2K1KKR9 | Y | Y *(Low)* | Y *(Low)* | **N** |

**References:**

Caspy, I., Borovikova-Sheinker, A., Klaiman, D., Shkolnisky, Y., Nelson, N. (2020) The structure of a triple complex of plant photosystem I with ferredoxin and plastocyanin. *Nat. Plants* **6**, 1300–1305. <https://doi.org/10.1038/s41477-020-00779-9>

Gorski, C., Riddle, R., Toporik, H., Da, Z., Dobson, Z., Williams, D., Mazor, Y. (2022) The structure of the *Physcomitrium patens* photosystem I reveals a unique Lhca2 paralogue replacing Lhca4. *Nat. Plants* **8**, 307–316. <https://doi.org/10.1038/s41477-022-01099-w>

Laughlin, T.G., Savage, D.F., Davies, K.M. (2020) Recent advances on the structure and function of NDH-1: The complex I of oxygenic photosynthesis. Biochim Biophys Acta (BBA) – Bioenerg. **1861**, 148254. <https://doi.org/10.1016/j.bbabio.2020.148254>

Su, X., Cao, D., Pan, X., Shi, L., Liu, Z., Dall'Osto, L., Bassi, R., Zhang, X., Li, M. (2022) Supramolecular assembly of chloroplast NADH dehydrogenase-like complex with photosystem I from *Arabidopsis thaliana. Mol. Plant.* **15**, 454–467. <https://doi.org/10.1016/j.molp.2022.01.020>
